# Fostering learning among the next generation of veterinarians: incorporating one health and antimicrobial stewardship into veterinary medicine training curricula in Ethiopia

**DOI:** 10.3389/fvets.2025.1690392

**Published:** 2026-01-06

**Authors:** Ndungu Nyokabi, Lisette Phelan, Stefan Berg, Johanna Lindahl, Lilian Korir, Bernard Bett, Emmanuel Muunda, Adane Mihret, Henrietta Moore, James Wood

**Affiliations:** 1 Institute for Global Prosperity, University College London, London, United Kingdom; 2 University of Edinburgh Business School, Edinburgh, United Kingdom; 3 School of Sustainable Food and Farming, Harper Adams University, Newport, United Kingdom; 4 Bernhard Nocht Institute for Tropical Medicine, Hamburg, Germany; 5 International Livestock Research Institute (ILRI), Nairobi, Kenya; 6 Department of Medical Biochemistry and Microbiology, Uppsala University, Uppsala, Sweden; 7 Department of Clinical Sciences, Swedish University of Agricultural Sciences, Uppsala, Sweden; 8 Lincoln Institute for Agri-Food Technology, University of Lincoln, Lincoln, United Kingdom; 9 Armauer Hansen Research Institute (AHRI), Addis Ababa, Ethiopia; 10 Department of Veterinary Medicine, University of Cambridge, Cambridge, United Kingdom

**Keywords:** zoonoses, biosecurity, AMR, veterinary curriculum, infection prevention and control

## Abstract

Universities play a crucial role in educating and training veterinarians, and their fostering of learning among the next generation of students is widely regarded as key to bringing about the cultural change required to realise a transition towards a ‘One Health’ approach to managing human, animal, and environmental health and underpin widespread adoption of antimicrobial stewardship practices. In Africa, there is a paucity of studies that have explored how veterinary training curricula are influencing veterinary medicine students’ perceptions of ‘One Health’ and the importance of antimicrobial stewardship. This study takes Ethiopia as a case study and explores how veterinary medicine students’ training influences perceptions regarding the benefits of taking a ‘One Health’ approach to managing public health risks and adhering to rational antimicrobial prescription practices and drug use. Data for this study were collected through an online questionnaire survey administered to 154 veterinary students at universities across Ethiopia. We found that the veterinary students were interested in receiving training related to the ‘One Health’ concept and indicated that such training would increase the likelihood that, upon graduation, they would be competent practitioners who could collaborate with other health sectors practitioners in addressing public health challenges. The students perceived a gap in the veterinary training curricula regarding rational antimicrobial prescribing and drug use; this is a concern given that antimicrobial resistance is an emerging problem in Ethiopia and worldwide. The results of this study underscore that veterinary training curricula play a key role in shaping students’ mindsets and practice, and that the provision of information, interdisciplinary and transdisciplinary practical training, and mentorship is key to fostering the learning required to ensure that students are holistic practitioners with the knowledge and capacity to implement at ‘One Health’ approach and antimicrobial stewardship in the future careers. The results highlight the imperative and opportunity for higher education institutions, particularly universities, and policymakers to ensure that national veterinary curricula are cognisant of and aligned with emerging approaches—such as the One Health approach, which advocates for interdisciplinary and multidisciplinary collaboration and communication - to managing the risks posed by infectious diseases to public health.

## Introduction

Globally, there is growing concern about the risk posed by emerging infectious diseases of animal origin to human health and the potential for epidemics or pandemics (1). Equally, there is concern about the extent to which antimicrobial overuse and misuse have led to antimicrobial resistance (AMR), which constitutes a major threat to human and animal health (2). Dealing with the threat posed by zoonoses and AMR to human and animal health necessitates collaboration between medical, veterinary, public health, environmental health and social science professionals (1) and the adoption of a holistic ‘One Health’ (OH) approach to safeguarding public health that can be defined as an integrated approach to addressing the factors contributing to the prevalence among and impact of infectious diseases of animal origin (zoonoses) on humans, and recognises that there is an imperative to engage in problem-solving at the interface of human, animal, plant, and ecosystem health (3, 4). Higher education institutions have played a crucial role in translating the concept of OH into practice through their provision of interdisciplinary training (4). However, although progress has been made in bridging the separation that has traditionally characterised human, environmental and animal health education and training systems, a divide still exists between general medical practitioners (GPs) and veterinarians (5) and there is continued limited understanding of how a OH approach to safeguarding public health can be practically incorporated into routine clinical settings (1).

The emergence and re-emergence of zoonoses has led to calls for higher education institutions to encourage greater levels of interprofessional communication and collaboration and, in particular, equip veterinarians with the knowledge, skills, expertise, and capacity to effectively respond and contribute—through a holistic, multi-disciplinary OH approach—to zoonoses threats and public health challenges faced (1). There is an imperative to break down the “silo profession-centric academic training,” whereby students are taught to be specialists in one field without being taught to collaborate with other practitioners in adjacent fields (1, 6, 7). As the future primary ‘frontline’ responders confronted with zoonotic diseases and sentinel events on a daily basis, veterinarian students should be encouraged and have the opportunity to engage and work with, for example, with environmental, social and medical sciences (8, 9). They should be introduced to the concept of OH at an early stage in their education; this will cultivate interdisciplinary thinking and also prepare them to collaborate as veterinarians with physicians and environmental scientists, as envisaged by the OH paradigm (5).

Although there is growing recognition of the benefits of a OH approach to managing public health challenges and the concept of rational drug use has also gained support across a range of disciplines, training opportunities—for professionals seeking to implement a OH approach and/or rational drug use in their daily work—remain limited (4). Veterinary training curricula have traditionally focused on imparting theoretical knowledge over showcasing how concepts such as OH might be put into practice due to time constraints and a lack of funding (for curriculum development and delivery); this has constrained the implementation of OH training (4, 10). To date, there has been little appreciation of the importance of integration, coordination, collaboration, and cross-fertilisation of ideas among human and animal health education disciplines, which has made it difficult to address endemic and emerging zoonotic diseases in a livestock production context (5). A lack of training regarding the rational use of drugs in veterinary medicine has adversely impacted livestock production, reduced the efficacy of drugs administered, increased the incidence of adverse effects of drugs, increased the risk of drug residues entering the food system, and contributed to the development of antimicrobial drug resistance (2).

In Africa, efforts have been made to build or strengthen multi-sectoral relationships at academic, government and inter-governmental levels, but progress as regards implementing a OH approach and rational drug use in the context of managing the risks of zoonotic diseases has nevertheless remained slow at the clinical practitioner level, in the absence of interprofessional communication, cooperation and collaboration among human, animal, and environmental health professionals (1, 11–14). The Africa One Health University Network (AFROHUN), the Capacitating One Health in Eastern and Southern Africa (COHESEA), One Health Central and East African (OHCEA) University Network, the One Health Research, Education and Outreach Centre in Africa (OHRECA) projects, led by the International Livestock Research Institute (ILRI), have focused on transforming the veterinary training and learning environment in African universities, through capacity building, collaborative curriculum design, and peer-to-peer benchmarking (12, 13).

This study takes Ethiopia as a case study, a country that has experienced a rapid growth in the number of universities offering veterinary medicine training (15) and mainstreaming OH competencies into their training curricula, establishing OH centres, and integrating OH courses into Doctor of Veterinary Medicine programs. For example, Addis Ababa University and Jigjiga University are now offering MSc programs in One Health-related fields (16, 17). To the best of our knowledge, however, a paucity of studies has explored how veterinary medicine students’ training in Ethiopia is influencing their perceptions regarding the benefits of taking a One Health approach to managing the risk posed by emerging infectious diseases to public health and adhering to rational antimicrobial prescribing practices and drug use. Responding to this knowledge gap, this study explores the knowledge and attitudes of veterinary medicine students in Ethiopia regarding OH and antimicrobial stewardship training. This paper contributes to the existing literature on veterinary medicine training, OH, AMR, and antimicrobial stewardship. It provides insights into how improved veterinary medicine curricula and training could equip veterinary professionals to work beyond traditional disciplinary silos and leverage their expertise alongside that of other disciplines (human medicine, social sciences, and environmental sciences) to prevent, detect, and respond to complex public health challenges, such as zoonoses.

## Methodology

This study was undertaken in Ethiopia and covered veterinary medicine students (3rd to 6th year students) studying at universities across Ethiopia. Data was collected from the students through an online survey. The survey was designed following a literature review of peer-reviewed papers on OH, antimicrobial resistance and antimicrobial stewardship training in veterinary medicine universities (17–23). The survey was pre-tested with five veterinary students (3 male and 2 female students) in their final year of study, and adjustments and corrections were made based on their feedback. The survey contained closed-ended questions focused on demographic characteristics (year of study, age, gender, etc.) and attitudes and knowledge relating to OH and antimicrobial stewardship. The survey was hosted on Survey Monkey^1^ for 7 weeks. An email link to the survey was shared with veterinary medicine students through their student associations, class representatives and organisations. Moreover, the survey link was distributed with the help of practising veterinarians and student organisations who shared it on social media platforms often used by veterinary medicine students. The survey was designed as an opt-in survey, whereby taking part was considered as consent and due to ethical considerations, no personal data was collected. The inclusion criteria for this study were that students were in their third year or above, had undertaken advanced practical microbial and epidemiology modules and had completed an internship placement. In the case of any individual who attempted to complete the survey but did not fit these inclusion criteria, the survey was automatically ended based on an initial set of demographic questions.

### Data management and analysis

In total, 300 students started to fill out the survey; however, incomplete entries were removed during data cleaning, resulting in 154 completed surveys. The data collected during the survey was downloaded as an Excel document, cleaned and analysed, with descriptive statistics (means and proportions) generated, using R statistical software.

## Results

The demographic characteristics of the 154 Doctor of Veterinary Medicine (DVM) students who participated in this study are summarised in Table 1. These students were pursuing degree programmes at 11 universities in different federal regions of Ethiopia. The majority of participating students were male (66%). Moreover, the majority of respondents were in their fifth year of studies (51%).

**Table 1:** Characteristics of participant veterinary students in this study (*n* = 154).

Average age	Years (mean ± SD)	24.8 ± 1.3
Gender (*n*/%)	Male	102 (66%)
	Female	52 (34%)
Year of study (*n*/%)	3	22 (14)
	4	26 (17)
	5	79 (51)
	6	27 (18)
University (*n*/%)	Addis Ababa University	21 (14)
	Gambela University	1 (0.6)
	Gonder University	34 (22)
	Haremaya University	21 (14)
	Hawassa University	7 (4.5)
	Jijiga University	6 (3.9)
	Jimma University	5 (3.2)
	Mekelle University	3(1.9)
	Samara University	5 (3.2)
	Wolita Sodo University	25 (16)
	Wollega University	26 (17)

In response to a question about their understanding of OH, 43 students (27.9%) indicated they conceptualised OH as a collaborative approach towards achieving the highest attainable standard of health, well-being, and equity worldwide through judicious attention to the human systems—political, economic, and social—that shape the future of humanity. Another subset of the respondents, 48 students (31.2%), thought it was a collaborative approach of multiple health science professions aimed at designing and implementing health programmes, policies, legislation and research to achieve better public health outcomes. The final subset of respondents, 63 students (40.9%), thought it was a collaborative framework involving the health of humans, animals, and ecosystems that aimed to ensure environmental sustainability and socioeconomic stability.

The veterinary students were asked about their interest in OH training (Table 2). The majority of students responded that they were interested in receiving training related to OH and interdisciplinary learning, partaking in student exchange programmes, networking within and beyond their discipline and community, as well as accessing internships and mentorship by professionals who were not veterinarians. However, one quarter of students (24%) were not interested in receiving OH training.

**Table 2:** Veterinary students’ interest in One Health training (*n* = 154).

How interested are you in the following?	Very interested (*n*/%)	Somewhat interested (*n*/%)	Minimally interested (*n*/%)	No interest (*n*/%)
One Health educational experiences	90 (58.4)	26 (16.9)	37 (24)	1 (0.6)
Lectures and courses taught by physicians	79 (51.3)	37 (24.0)	37 (24.0)	1 (0.6)
Lectures and courses taught by ecologists	84 (54.5)	34 (22.1)	35 (22.7)	1 (0.6)
Lectures and courses taught by biologists	77 (50.0)	46 (29.9)	27 (17.5)	4 (2.6)
Lectures and courses taught by an interdisciplinary team of health professionals	74 (48.1)	49 (31.8)	25 (16.2)	6 (3.9)
Student exchange programmes with human health medical colleges	60 (39.0)	64 (41.6)	25 (16.2)	5 (3.2)
Student exchange programmes with public health schools	58 (37.7)	66 (42.9)	24 (15.6)	6 (3.9)
Research opportunities with nonveterinary ‘One Health’ practitioners	60 (39.0)	66 (42.9)	24 (15.6)	4 (2.6)
Networking opportunities with nonveterinary ‘One Health’ practitioners	57 (37.0)	61 (39.6)	28 (18.2)	8 (5.2)
Interdisciplinary professional conferences for veterinarians, physicians, and ecosystem health experts	61 (39.6)	54 (35.1)	33 (21.4)	6 (3.9)
Health internships hosted by faculties other than those within the Colleges of Veterinary Medicine and Biomedical Sciences	64 (41.6)	46 (29.9)	34 (22.1)	10 (6.5)
Access to ‘One Health’ mentors who are non-veterinarians	70 (45.5)	44 (28.6)	34 (22.1)	6 (3.9)

Table 3 presents veterinary students’ perceptions and opinions regarding the promotion of the OH approach and related training in the veterinary medicine curriculum. The majority of students thought that OH was being promoted in the veterinary sector in Ethiopia and regarded national and international efforts as commendable, particularly the focus on disease control and surveillance. However, they took the view that more efforts should be put into strengthening OH training in veterinary curricula, focusing on the human-animal-environment interface. In their DVM degree programmes, students opined, they were not receiving training on the OH approach, how to collaborate with other healthcare professionals, and how to interact with and communicate with farmers.

**Table 3:** DVM students’ perceptions and opinions regarding the promotion and training of the One Health approach in Ethiopia (*n* = 154).

	Yes (*n*/%)
Is the ‘One-Health’ approach adopted in your field?	134 (87.0)
Do you think veterinary medicine has a role to play in preventing human diseases?	147 (95.5)
‘One-Health’ implies better cooperation between doctors and veterinarians	142 (92.2)
‘One-Health’ means working on the interaction between human and animal health and the ecosystem	108 (70.1)
‘One-Health’ is a new approach to global health governance	69 (44.8)
Is ‘One Health’ an important approach for you?	73 (47.4)
Have you applied the ‘One Health’ approach in your work/projects/training?	64 (41.6)
Should Ethiopia organise more events and communicate more to spread and implement the ‘One Health’ approach?	80 (51.9)
Should Ethiopia put in place ‘One Health’ national strategies and coordination structures?	110 (71.4)
Should Ethiopia strengthen cooperation with international organisations (WHO / FAO/ OIE)?	104 (67.5)
Should Ethiopia focus on specific diseases that develop at the human-animal-environmental interface?	95 (61.7)
Knowledge and skills in communicating about zoonoses and human health	
I am being trained to work with human health workers	137 (89.0)
I am being trained to communicate with human health workers	139 (90.3)
I would be able to recognise if a farmer is affected by a zoonosis	145 (94.2)
I would advise farmers to seek medical care when they realise their animals have an important zoonotic disease	130 (84.4)
I am concerned about the health of farmers when treating their animals	103 (66.9)
I am concerned about my health when treating sick animals	101 (65.6)

Students’ perceptions and attitudes towards OH and inter-professional collaboration were also explored (Table 4). Most students agreed that the OH approach would likely shape how the veterinary sector operated in Ethiopia in the future. However, less than half of the students felt that they had received sufficient training on personal protection, antimicrobial guidelines, and antimicrobial stewardship, given the occupational risks associated with exposure to zoonoses. Furthermore, the students felt that they had not been properly trained to advise farmers on zoonoses and biosecurity measures, and how they could adapt the production strategies to protect themselves from zoonoses.

**Table 4:** Students’ perceptions and attitudes towards OH and inter-professional collaboration.

	Agree (*n*/%)	Neutral (*n*/%)	Disagree (*n*/%)
How do you feel about the following ‘One Health’ statements?			
‘One Health’ is an important approach that will shape the veterinary/animal health profession	119 (77.0)	35 (23.0)	0 (0.0)
As a veterinarian, I must promote the ‘One Health’ approach	117 (76.0)	37 (24.0)	0 (0.0)
There are enough practical frameworks for veterinarians to follow or promote issues about ‘One Health	117 (76.0)	32 (20.8)	5 (3.2)
My contribution to control/treat zoonoses must bring about a good outcome for the animal	116 (75.3)	37 (24.0)	1 (0.6)
My contribution to control/treat zoonoses must bring about a good outcome for the farmer	95 (61.7)	59 (38.3)	0 (0.0)
I have a good understanding of protecting myself and fellow staff from potential zoonoses risks	76 (49.4)	75 (48.7)	3 (1.9)
I have a good understanding of antimicrobial stewardship guidelines for veterinarians	70 (45.5)	80 (51.9)	4 (2.6)
What is your opinion regarding animal-human health inter-professional collaboration?			
Veterinarians are better equipped and more knowledgeable than physicians in understanding and approaching zoonotic cases	103 (66.9)	49 (31.8)	2 (1.3)
Having a human health referral system to consult veterinarians’ animal-related knowledge would bring about positive changes to human health	100 (64.9)	53 (34.4)	1 (0.6)
I would be willing to collaborate with physicians to manage zoonotic cases that affect both my animal patients and human clients	101 (65.6)	53 (34.4)	0 (0.0)
I expect to be knowledgeable enough, in the future, to provide advice to human clients about preventing zoonotic diseases commonly transmitted from livestock and companion animals	100 (54.9)	51 (33.1)	3 (1.9)
It will be an important part of my future work to provide advice to clients about preventing zoonotic diseases commonly transmitted from livestock and companion animals	87 (56.5)	62 (40.3)	5 (3.2)
A veterinarian should always ask clients if there are any immune-compromised and immune-deficient members, pregnant, young or elderly members living with the livestock as part of the basic information	78 (50.6)	71(46.1)	5 (3.2)

Table 5 presents the students’ attitudes towards antimicrobial prescribing practices and stewardship. A significant proportion of the students did not perceive AMR as a problem in Ethiopia, and for the veterinary sector. There was a low perception of the role that veterinarians’ drug prescription could play in contributing to AMR. A significant number of students, however, conceded that there was misuse of antimicrobials in veterinary practice.

**Table 5:** The DVM students’ perceptions towards AMR in Ethiopia (*n* = 154).

		*n* (%)
To what extent is AMR a problem in Ethiopia?	Serious problem	86 (55.8)
	Moderate problem	15 (9.7)
	Minor problem	45 (29.2)
	Not at all a problem	8 (5.2)
To what extent do you think addressing AMR is currently a priority in Ethiopia?	Not a priority	25 (16.2)
	Low priority	88 (57.1)
	Medium priority	18 (11.7)
	High priority	18 (11.7)
	Essential	5 (3.2)
If AMR is a problem, what do you think is influencing antimicrobial resistance in Ethiopia?	Antimicrobial prescribing behaviour	76 (49.4)
	Misuse of antimicrobials	125 (81.2)
	Non-observation of withdrawal periods	118 (76.6)
	Use of antimicrobials in feeds	122 (79.2)
	Symptomatic treatment of animals (without lab results and guidance)	94 (61.0)
	Sub-standard drugs	80 (51.9)
	Overuse of drugs	42 (27.3)

Table 6 presents students’ personal beliefs about antimicrobial use and AMR. The results indicate knowledge and perception concerning the appropriate use of antimicrobials and their impact on animal health and livestock production. A significant percentage of students were neutral in their opinions, which could imply limited knowledge of the issues or that they did not perceive antimicrobial use and AMR as important topics in the field of veterinary medicine.

**Table 6:** Students’ personal beliefs about the usage of antimicrobial resistance (AMR) (*n* = 154).

	Agree (*n*/%)	Neutral (*n*/%)	Disagree (*n*/%)
AMR is a consequence of antimicrobial misuse and overuse nationally	126 (81.8)	26 (16.9)	2 (1.3)
Better use of antimicrobials will reduce problems with AMR	121 (78.6)	32 (20.8)	1 (0.6)
Knowledge of the appropriate use of antimicrobials is important in my veterinary career	123 (79.9)	30 (19.5)	1 (0.6)
I would like more education on AMR	107 (69.5)	44 (28.6)	3 (1.9)
New antimicrobials will be developed in the future that will keep up with the problem of AMR	93 (60.4)	57 (37.0)	4 (2.6)
Poor infection control practices by veterinary professionals cause the spread of AMR	80 (51.9)	70 (45.5)	4 (2.6)
Inappropriate use of antimicrobials causes AMR	73 (47.4)	74 (48.1)	7 (4.5)
AMR will affect animal health and production	75 (48.7)	72 (46.8)	7 (4.5)
I have sufficient knowledge of antimicrobial use for future clinical practice	87 (56.5)	59 (38.3)	8 (5.2)
Antimicrobials used to treat animals can remain within their tissues	93 (60.4)	54 (35.1)	7 (4.5)

With regard to which antimicrobial to prescribe to an animal, the students were asked about the sources from which they received information and their importance (Table 7). Students indicated that they tended to rely on training and knowledge gained through the veterinary medicine curriculum. However, they also looked to source additional information, particularly from online sources. The shift to online sources of information was driven by increased access to mobile phones and the internet.

**Table 7:** Information sources and their importance for antimicrobial prescription among DVM students (*n* = 154).

	Not at all important (*n*/%)	Slightly important (*n*/%)	Moderately important (*n*/%)	Very important (*n*/%)	Extremely important (*n*/%)
Pharmaceutical company representatives	30 (19.5)	34 (22.1)	29 (18.8)	46 (29.9)	15 (9.7)
Label or package inserts	20 (13.0)	31 (20.1)	35 (22.7)	46 (29.9)	22 (14.3)
Peer-reviewed scientific literature	10 (6.5)	34 (22.1)	37 (24.0)	48 (31.2)	25 (16.2)
Peers within my practice/office	9 (5.8)	33 (21.4)	34 (22.1)	41 (26.6)	37 (24.0)
Peers beyond my practice/office	10 (6.5)	31(20.1)	38 (24.7)	38 (24.7)	37 (24.0)
Clinicians and pharmacists	9 (5.8)	24 (15.6)	35 (22.7)	48 (31.2)	38 (24.7)
Veterinary Information Networks (VIN)	8 (5.2)	25 (16.2)	36 (23.4)	50 (32.5)	35 (22.7)
Online resources (e.g., blogs, media posts or web searches)	5 (3.2)	25 (16.2)	37 (24.0)	63 (40.9)	24 (15.6)
Textbooks or drug handbooks	6 (3.9)	18 (11.7)	44 (28.6)	56 (36.4%)	30 (19.5)
Applications on a smartphone or tablet (Phone Apps)	5 (3.2)	25 (16.2)	45 (29.2)	42 (27.3)	37 (24.0)
Online formulary (list of medicines)	12 (7.8)	16 (10.4)	50 (32.5)	50 (32.5)	26 (16.9)

Table 8 presents students’ perception of their competence gained through training and their readiness to transition to practice after graduation. The students thought they had good theoretical skills but felt the need to improve their practical and interpersonal skills. A significant proportion of students (approximately 25%) expressed that they were not yet confident in using the skills learned through training, such as how to deal with demanding clients.

**Table 8:** Students’ perception of their competence gained through training and their readiness to transition to practice after graduation (*n* = 154).

	Completely confident (*n*/%)	Fairly confident (*n*/%)	Somewhat confident (*n*/%)	Slightly confident (*n*/%)	Not at all confident (*n*/%)
To know when to start antimicrobial therapy	34 (22.1)	22 (14.3)	25 (16.2)	69 (44.8)	4 (2.6%)
How to select the best antimicrobial drug for a specific infection	33 (21.4)	26 (16.9)	26 (16.9)	64 (41.6)	5 (3.2)
How to select the correct dosing	34 (22.1)	21 (13.6)	37 (24.0)	58 (37.7)	4 (2.6)
How to select the right duration of treatment for specific infections	29 (18.8)	24 (15.6)	45 (29.2)	50 (32.5)	6 (3.9)
To describe the correct spectrum of antimicrobial therapy for different antimicrobials (what is covered by each drug)	28 (18.2)	31 (20.1)	51 (33.1)	32 (20.8)	12 (7.8)
Understand the basic mechanisms of antimicrobial resistance	24 (15.6)	40 (26.0)	57 (37.0)	20 (13.0)	13 (8.4)
How to streamline or de-escalate antimicrobial therapy	22 (14.3)	45 (29.2)	56 (36.4)	19 (12.3)	12 (7.8)
How to interpret antibiogram	18 (11.7)	45 (29.2)	51 (33.1)	30 (19.5)	10 (6.5)
How to find reliable sources of information to treat infections	24 (15.6)	43 (27.9)	43 (27.9)	32 (20.8)	12 (7.8)
How to handle a client who demands a particular antimicrobial therapy	31 (20.1)	36 (23.4)	47 (30.5)	25 (16.2)	15 (9.7)

## Discussion

This study explored the knowledge, attitudes and training of veterinary medical students regarding OH and antimicrobial stewardship in Ethiopia. The results of this study underscore that better educating future veterinary practitioners could significantly contribute to the implementation of the OH approach, the reduction of antimicrobial misuse, and the prevention of the rise of AMR (18, 20). Veterinary education in Ethiopia has been characterised by a focus on theoretical knowledge due to a lack of facilities suited to practical training (2, 15). Additionally, similar to other countries in Africa, information concerning education programmes and the quality of teaching by academic staff is scarce or non-existent (15, 24, 25). Veterinary medicine training course quality and content have not been assessed across Africa as a whole, nor in individual countries such as Ethiopia (2, 15, 24, 26).

### Students’ interest in OH and antimicrobial stewardship in veterinary training

The results of this study indicate that DVM students are interested in OH training and antimicrobial stewardship, and believe that this training would prepare them to be veterinary medicine practitioners with the skill set required to collaborate with human health practitioners (20, 27, 28). The concept of OH, in particular, was perceived by students as having the potential, if correctly implemented, to change how veterinary medicine was practised in Ethiopia. The findings suggest the imperative to adapt veterinary training curricula to place greater emphasis on collaboration and coordination between social and behavioural scientists, and human, environmental and animal health practitioners (20, 29). However, one quarter of the students that participated were not interested in OH training; this could be reflective of the traditional siloed culture of disciplinary training that has long characterised veterinary training and underscores the challenge that educators may face as regards fostering a collaborative mindset among students. Information, interdisciplinary and transdisciplinary training, and mentoring, will be key to helping students recognise and understand that addressing human and animal health challenges requires a holistic systems approach and collaboration and communication with practitioners beyond their own discipline (15, 20).

### Currently knowledge gaps in the veterinary medicine curricula regarding OH and antimicrobial stewardship

The findings of this study reveal that students had limited knowledge of OH as an approach to managing public health challenges, such as zoonotic outbreaks, and a limited perception of the links between drug prescription and misuse of antimicrobial and AMR. This could be due to students’ limited exposure to real-world practices. Studies indicate that practising veterinarians recognise AMR as a serious concern and a major constraint to veterinary services (18, 20). This suggests that Ethiopian universities with DVM degree programmes could contribute to bridging the current theory-practice gap by integrating real-life cases into their veterinary medicine curriculum (17, 18, 20). There is an imperative to educate veterinary students about the benefits of antimicrobial stewardship, prudent prescription of antimicrobials, and the impacts of antimicrobial misuse on animal health and production (30). Students should be equipped with the practical and interpersonal skills required to explain to and convince farmers of best practices and/or alternative treatment options and graduate from DVM degree programmes with both techne (scientific knowledge) and metis (experiential knowledge) (15, 31–33).

Veterinary students’ low perception of the risk of AMR is in agreement with previous studies (18, 34). Knowledge gaps highlighted in this study are similar to the findings of Fasina et al., who conducted a multi-country survey of veterinary students from Nigeria, South Africa, and Sudan (18). Students’ limited knowledge of OH could be due to the overlap in the definitions and scope of concepts similar to OH, namely, Ecohealth, and Planetary Health (24, 28). Similar to OH, Ecohealth can be conceptualised as a systems-based approach to promoting health and well-being, however, it has a stronger focus on social and ecological interactions (35). Planetary health is premised on the recognition of our planetary limits and is an inclusive approach focused on social dimensions of health (36, 37).

### Importance of training and increasing students’ access to reliable sources of information regarding OH and antimicrobial stewardship

The results of this study highlight low knowledge of the risks associated with zoonoses among DVM degree students in Ethiopia; this should be of concern to educators and policymakers alike, given the prevalence of endemic zoonoses that can affect veterinary professionals’ personal health status and, by extension, their ability to contribute to safeguarding public health (26, 38, 39). Incorporation of the OH concept into veterinary medicine curricula will increase the likelihood of students—the next generation of veterinary practitioners—understanding that environmental, human and animal health are connected and that the epidemiology and ecological drivers of zoonoses should be addressed holistically and through an interdisciplinary lens (2, 20, 27, 40). This will ensure that they are in a position to advise farmers and clients on zoonoses and biosecurity measures that can be adopted to protect both themselves and consumers; this will contribute to reducing the zoonoses burden in Ethiopia (24, 26). Veterinarians play an crucial role in encouraging farmers to adopt preventive measures and improve their animal health-seeking behaviour and make an important contribution to positive public health outcomes through their work, notably, by providing animal health services, biosecurity advice, and conducting food safety inspections (6, 26, 39). Students should thus be encouraged to implement the principles of OH in their day-to-day activities upon graduation (15, 27).

Low knowledge levels of antimicrobial guidelines and poor perceptions regarding the relationship between antimicrobial stewardship and AMR among students should also concern educators in Ethiopia, given that the antimicrobial prescription behaviour of veterinarians directly contribute to antimicrobial misuse and AMR (2, 20). The results of this study highlight an urgent need to educate veterinarian students about the crucial role that they can play in disseminating information about responsible antimicrobial use to farmers and increasing awareness about regularly updated antimicrobial use guidelines, understanding of the role of good biosecurity and vaccination practices in disease prevention, and availability of laboratory services at affordable costs (2, 40). Changing veterinarian students’ mindsets as regards the impact of their drug prescription behaviour and provision of training, education, and information to farmers could better prepare them to understand and address the magnitude of the AMR problem in Ethiopia (2, 26).

The findings of this study indicate that veterinary students in Ethiopia are sourcing information and deriving knowledge regarding zoonoses from online sources; this reflects technological changes and increased access to smartphones and growing internet use among the younger strata of society and a willingness to access information and learn from what is happening globally in a public health context (2, 15, 38–41). There is, however, a need to ensure information sources are credible, promote best practices, and help students make better decisions once they graduate as veterinarians (30, 39, 41). Online information sources should be leveraged to improve the training experience for DVM degree students who could benefit from completing Massive Open Online Courses (MOOCs) relating to OH, antimicrobial stewardship, and AMR (38, 41).

### Policy implications

The results of this study are promising for the future of veterinary medicine and the implementation of the OH approach in Ethiopia, highlighting DVM degree students’ awareness of and interest in OH as an approach to safeguarding public health. The policy implication of the findings is that, beyond adapting the veterinary medicine curricula to incorporate the OH concept to a greater extent, there is an imperative to establish the institutional structures required to ensure that OH is implemented across the health sector and ensure that there is adequate funding to realise a coordinated, interdisciplinary approach to managing the risk posed by zoonoses, ensure access to high quality animal and human health services, and securing positive environmental, human and animal health outcomes (4, 6, 17, 28). In Ethiopia, as in other African countries, OH is an approach that could increase coordination and collaboration and help practitioners overcome the entrenched silo culture of working within the health sector (6, 17, 28).

In contrast, the findings of this study suggest a knowledge gap exists related to rational antimicrobial prescription and AMR, despite increasing awareness that prescription practices for veterinary drugs are a problem in Ethiopia. Irrational drug use has been attributed to the low availability of diagnostic facilities and the absence of a drug formulary and standardisation of veterinary treatment guidelines (2, 42). There is an imperative for policymakers and educators to ensure that veterinary students are aware of the importance of rational antimicrobial prescription practices and use (2, 20, 33, 42). Veterinary educational programmes should go beyond purely theoretical training to include practical training that equips students with real-life knowledge and experience (15, 43). Regular course quality and content assessment could contribute to ensuring continuous improvement of the veterinary curricula, and ensure emerging trends become part of veterinary training in Ethiopia (15, 20).

In Ethiopia, as in other African countries, there has been a concerted effort at policymaking, academic, and civil society levels to learn from global and local initiatives aimed at improving the provision of health care (2, 6, 17, 20). The results of this study underscore the scope to establish better contacts between Ethiopian universities with other universities in Africa and globally, to facilitate collaboration and cross-sectoral learning (15, 20). There are opportunities to build on existing and emerging One Health coordination structures that exist in Ethiopia (17). There are various ongoing multi-stakeholder OH Initiatives and collaborations between the government, academic, research institutions, NGOs, and donor organisations that aim to transform animal, human and environmental health (16, 44). There is need to support national, regional and international networks and collaborations including: Jigjiga One Health Initiative (JOHI), funded by the Swiss Agency for Development and Cooperation (SDC); The Ohio Global One Health Initiative, led by the Ohio State University Health Sciences; the One Health Central and East African (OHCEA) University Network; the One Health Regional Network For the Horn Of Africa (HORN); The Africa One Health University Network (AFROHUN); the Capacitating One Health in Eastern and Southern Africa (COHESEA); the One Health Research, Education and Outreach Centre in Africa (OHRECA). These initiatives are all looking to transform training related to human, animal, and environmental health (12, 13, 16, 17). A shift in silo or disciplinary culture will be needed, however, to make OH approach and practice possible. The findings of this study highlight the need for greater information sharing and communication between practitioners in the animal, human and environmental health sectors, improvements as regards OH approach competencies and expertise at a sub-national level, and the importance of a formal or specific budget to underpin the implementation of OH plans and activities across Ethiopia (11, 16).

### Limitations of this study

The main limitation is the limited sample of the participants, which constrains wider population generalisation of the research findings. Future studies should focus on surveying a larger population sample size and university coverage to tease out contextual information and highlight the specific need for different universities’ curricula. Future studies should also undertake longitudinal studies that map the evolution of OH in Ethiopia and the wider East Africa region. Future studies can also evaluate the capacity development of students and explore their post-training experiences as practising vets.

## Conclusion

The findings of this study, which explored the knowledge and attitudes of veterinary medicine students regarding OH and antimicrobial stewardship training, suggest that information provision, interdisciplinary and transdisciplinary training, and mentoring could increase coordination and collaboration and contribute to breaking the silo culture characterising public health sector responses to zoonoses in Ethiopia. Currently, DVM degree programmes do not encourage veterinary students to conceptualise epidemiology and the ecological drivers of zoonoses through an interdisciplinary lens. The results of this study suggest it is imperative that universities—which will continue to play a crucial role in educating, training, and fostering learning among the next generation of veterinary practitioners—ensure students are in a position to access information sources that are credible and encourage integration, coordination, collaboration, and cross-fertilisation of ideas among human and animal health education disciplines. This will contribute to bringing about behaviour change among students who are interested in OH and, concurrently, those who lack appreciation for the links between drug prescription and misuse of antimicrobials and AMR, and underpin the cultural change required to realise a transition towards holistic management of human, animal, and environmental health and widespread adoption of antimicrobial stewardship practices.

## Data Availability

The raw data supporting the conclusions of this article will be made available by the authors, without undue reservation.

## Footnotes

1
https://uk.surveymonkey.com
